# Effects of Prolonged Irriadiation (by X-Rays) of the Abdomen

**Published:** 1913-04

**Authors:** 


EFFECTS OF PROLONGED IRRIADIATION (BY X-RAYS) OF THE ABDOMEN. Regaud, Nogier and Lacassagne, *Arch. d'Elect. Med.*, October 11, 1912, report experiments, mainly on dogs, with protocols, illustrations of pathologic histology, etc. Applications of an hour or two in duration, rather constantly caused death in one or a few days. The rays were filtered so as to avoid their being too much absorbed by the skin and abdominal wall. They found that the superficial gastric epithelium was not very sensitive, neither were the pyloric glands but the glands of the fundus showed atrophy, especially of the chief cells. The intestinal villi were denuded at their tips and the crypts of Lieberkühn showed even more extensive necrobiosis and absorption of cells. The lymphoid and connective tissues were not particularly affected. A difference of degree was noted according to the distance of loops of intestine from the radiant source. The various lesions predispose to preforation. They call attention to the special danger of filtered rays from powerful tubes, the point of danger being shifted from the superficial to the visceral structures. Therapeutic applications, as in cancer, and the accidental exposure of operators, extending over a long period, they consider especially dangerous.

